# Morbidity Patterns and Health Care Seeking Behavior among Older Widows in India

**DOI:** 10.1371/journal.pone.0094295

**Published:** 2014-04-09

**Authors:** Gopal Agrawal, Kunal Keshri

**Affiliations:** 1 International Institute for Populations Sciences, Mumbai, India; 2 G. B. Pant Social Science Institute, Allahabad, India; Oregon Health & Science University, United States of America

## Abstract

In the process of health transition, India is facing rapid pace of demographic aging. Rapid increase in older adult population posed serious concerns regarding health and health care utilization for them. However, very limited research documented resultant implications of demographic aging for health and health care use in the nexus of marital status and gender. With this perspective, the present study examined patterns in morbidity prevalence and health seeking behaviour among older widows in India. Multivariate logistic regression models were estimated to examine the effects of socio-demographic conditions on morbidity prevalence among older widows and their health care seeking behavior. Data from the latest 60^th^ round of National Sample Survey (NSS), 2004 was used. Overall, morbidity prevalence was 13% greater among older widows compared to older widowers. Adjusted prevalence of communicable and non-communicable diseases was found 74 and 192 per 1000 older widows respectively. At the same time, likelihood of seeking health care services for reported morbidities was substantially lower among older widows. The findings of this study are important to support policy makers and health care providers in identifying individuals ‘at risk’ and could be integrated into the current programs of social, economic and health security for the older persons.

## Introduction

The process of aging is lead by the decline in fertility reinforced by increasing longevity due to falling mortality in older ages [1]. It produces unprecedented changes in the age-structure of all societies, notably the historic reversal in the proportions of younger and older persons. In the turn of 21^st^ century, population aged 60 and above is increasing at an accelerated rate and most of the rapid growth is projected in developing countries including India [2]–[6]. In 2011, the share of older persons aged 60 years and above was 8.6% which placed India in ‘aged’ category as per United Nations classification [7]–[8]. Undoubtedly, the process of health transition has accelerated in India and consequently, India has to confront rapid pace of population aging [5], [9]–[10].

In the course of population aging, marital status composition requires special attention in the nexus of social and health concerns among older adults. The proportion of older widowed women has increased more rapidly compared with men leading to wider gender disparities in older ages [2], [5], [11]–[12]. In India, sex ratio among older adults (60+) showed that there are only 29 men for every 100 women. There are 19.6 million older widows in age 60 years and above [12]. Higher life expectancy among women, differences in the ages at which men and women marry as well as differing proportions of older men and women who remarry are the significant causes responsible for one-tailed skewed sex ratio among older adults [1]–[2], [11], [13]–[15].

In India, the size of older widows is not a small number especially when only few states viz. Kerala and Tamil Nadu are in advanced stages of demographic transition. This segment of population is growing swiftly with more and more states are progressing in advanced stages of health transition. At the same time, ageing is associated with greater reporting of morbidities and co-morbidities among older persons due to fragile and weakening immune system. The growing proportion and size of older widows thus poses critical challenges to tackle multiple problems of their health and well-being in India [2], [9], [13].

A review of past studies on aging in India guides us to proceed with the relevant theoretical perspective. Evidence suggests that women enjoyed higher life expectancies in the past centuries. Nevertheless, they were at greater risk of suffering with poorer health conditions than men, in all terms of self-rated health, functional status, physical functioning and greater immobility [16]–[23]. At the same time, likelihood of seeking health care services was higher for men than women among those reporting multiple morbidities or severe impairments [9], [20], [23].

Association of gender and marital status with health and health care utilization among older adults is well documented in previous studies [15], [24]–[27]. Widowhood has direct impact on psychological and physical health of older persons. Level of health care utilization is substantially lower among widowed elderly compared with married older persons [9], [28]–[37]. However, most studies quoted here are of developed countries. On the other hand, limited research on health conditions of older widows is carried out in developing countries including India and provides scarce information on health conditions of older widows particularly in terms of gender disparities.

It is well known fact that widows in India were often exposed to social neglect, sexual abuse, violence and isolation. Previous studies well documented that widows in India were underprivileged even for basic human needs of food, shelter and medical aids, forcing them to live with chronic ill-health conditions. However, no attempts have been made so far to study the disease patterns among older widows and their treatment seeking behavior. Given the large proportion of older widows, we focused to a) compare the patterns of disease prevalence among older widows in terms of communicable, non-communicable and other diseases, b) treatment seeking behavior of older widows c) study their variations by socio-economic and demographic factors.

## Materials and Methods

The present study used data from the latest 60^th^ round of National Sample Survey (NSS) conducted in 2004 on the subject of Morbidity and Health Care with the condition of aged persons (60+) in special focus. National Sample Survey is the only rich source of information on health and health care use in India. This survey was carried out in all the States and Union Territories of India.

Since its inception in 1950, the National Sample Survey is single large scale household survey in India. NSS periodically collects information on various social, economic, demographic and health aspects of population in India. In the 60th round of NSS survey, data was collected through a survey on the subject of ‘Morbidity and Health Care’. In the survey on Morbidity and Health Care, the following main aspects were covered: a) Morbidity and utilisation of health care services including immunisation and maternity care, b) Problems of aged persons, and c) Expenditure of the households for availing the health care services.

In this round, a total number of 73,868 households and 383,338 persons were surveyed at national level. Under this survey, special information was collected from the 34,831 persons aged 60 years and above about their social, economic, health and health care conditions. Out of the total older adult (60+) population, number of older widowers and widows respectively were 3,267 and 10,111. Our main analyses were focused on older widows and thus our sample size consists of a total number of 10,111 widows aged 60 years and above. In this study, analysis on 3,267 older widowers is very limited, just to have comparative picture of morbidity prevalence between older widowers and widows and to set rationales for the present study.

The prevalence rate for a specific disease was defined as the proportion of people reporting a disease or morbidity. In our study, it is expressed as the ratio of number of older widows/widowers reporting that specific disease to the total number of older widows/widowers exposed to the risk of reporting that specific disease multiplied by 1000. The reference period for the prevalence rate was the last 15 days prior to the survey.

Multivariate multinomial regression models were fitted to examine the influence of socio-economic and demographic factors on morbidity prevalence patterns in terms of communicable, non-communicable and other diseases. The dependent variable was measured on nominal scale and delineated in four categories: coded ‘1’ for reporting a communicable disease, ‘2’ for reporting a non-communicable disease, ‘3’ for reporting other diseases and ‘0’ for not reporting a disease. The “other diseases” category included accidents/injuries/burns/fractures/poisoning and other diagnosed and un-diagnosed diseases. It also included disabilities reported by the older adults.

The following multivariate multinomial regression models were estimated to assess the morbidity prevalence patterns by socioeconomic and demographic predictors of older widows. The mathematical form of the regression models fitted is given as below.
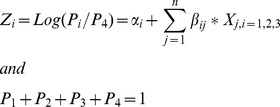
where,

α*_i,_ i* = 1,2,3: constants

β*_ij,_ i* = 1,2,3; *j* = 1,2,…. *n*: multinomial regression coefficient.

P_1_: Estimated probability of reporting a communicable disease by an older widow.

P_2_: Estimated probability of reporting a non-communicable disease by an older widow.

P_3_: Estimated probability of reporting any other disease by an older widow.

P_4_: Estimated probability of not reporting a disease by an older widow.

Here, P_4_ was the reference category.

In this way, regression coefficients were estimated by fitting and applying the above multivariate multinomial regression model. It is most important to note that the interpretation of multinomial regression coefficients is not straightforward as in the case of linear and logit regression models. For scientific reasons, one cannot simply interpret them in either forms of coefficients and odd ratios. The details justifications are published elsewhere [9], [38]. The most convenient way to present the results of multinomial logistic regression is to convert regression coefficients into adjusted percentages. The procedure consists of the following steps:

1. Step 1: By using regression coefficient and weighted proportions of population under study in each category of independent variables, the probability was computed as: 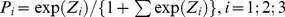 and P_4_ = 1−P_1_+ P_2_+ P_3_ where Z was the estimated value of response variables for all categories of each variable.
2. Step 2: The probability estimates obtained in step 1 were then multiplied by 100 to get adjusted morbidity prevalence rate (%).

In the model of utilization of health care services, dependent variable was measured on nominal scale and dichotomous in nature. Therefore, multivariate binary logistic regression model was fitted to assess the effect of socio-economic and demographic factors on the treatment seeking behavior among older widows. The dependent variable in this model was of binary response: coded ‘1’ if an older widow received treatment for the reported morbidities, otherwise coded ‘0’. The sample for this analysis included only those older widows who reported morbidities.

All the analyses in this paper were performed with STATA 11.0 software. Appropriate weights were applied in all the statistical operations.

### Categorization of Predictor Variables

Evidence available in previous literature suggested that morbidity prevalence among the older persons and their treatment seeking behaviour vary substantially by socio-economic factors. In accordance with evidence from previous literature, the predictor variables included in multivariate regression models were age, sex, residence, social group, religion, educational level, and economic independence, monthly per capita expenditure (MPCE) percentile class, and living arrangements of older widows. The grouping of these predictor variables were as described below. The basic description about the independent variables is given in Appendix Table S1.

#### Age

Age 70+, age 65–70, age 60–65 (Reference Category-RC)

#### Residence

Urban, rural (RC)

#### Social group

Scheduled caste (SC) & scheduled tribes (STs), other backward classes (OBCs), others (RC)

#### Religion

Muslim, Others, Hindu (RC)

#### Education

High school and above, middle school complete, <middle school complete, illiterates (RC)

#### Living arrangement

Living with children and other relatives, living with other non-relatives, living alone (RC)

#### Economic independence

Full dependent, partially dependent, not dependent (RC)

#### Monthly Per Capita Expenditure (MPCE) percentile class

class5, class4, class3, class2, class1 (RC).

## Results

### Morbidity Prevalence Among Widowed Older Adults

Table 1 presents morbidity prevalence rates per 1000 persons by various categories of diseases among older widowed persons (60+). Overall, morbidity prevalence was significantly greater among older widows (337) compared to older widowers (299) with a corresponding gender ratio of 1.13 (p<0.001). The prevalence of communicable diseases was lower among older widows (66) compared to older widowers (75) with a corresponding gender gap of 0.87 (p<0.001). On the other hand, the prevalence of non-communicable diseases was significantly greater among older widows (344) by 18% (p<0.001) compared to older widowers (292).

**Table 1:** Morbidity Prevalence (per 1000) Among Widowed Older Persons (age 60 and above) by Sex in India, 2004.

Morbidities^a^	Total	Male	Female	Gender gap@
**Communicable diseases**	**68**	**75**	**66**	0.87***
1. Diarrhoea/dysentery	5	3	6	1.75***
2. Fever of unknown origin	13	13	13	1.07
3. Tuberculosis	4	7	3	0.43**
4. Whooping cough	6	10	5	0.48**
5. Diseases of kidney/urinary system	4	8	3	0.37**
6. Diseases of Skin	6	9	5	0.58***
7. Gastritis/gastric or peptic ulcer	21	18	21	1.16*
8. Other communicable diseases	8	7	9	1.28**
**Non-communicable diseases**	**331**	**292**	**344**	1.18***
1. Cataract	52	54	51	0.94
2. Diseases of eye	13	9	15	1.56***
3. Disorders of joints and bones	92	85	95	1.11
4. Bronchial Asthma	35	43	32	0.75***
5. Mellitus Diabetes	27	17	30	1.70***
6.Respiratory including ear/nose/throat ailment	14	18	13	0.74
7. Mental disorder	16	12	17	1.48***
8. Heart Diseases	18	17	18	1.04
9. Hypertension	56	33	63	1.90***
10. Other non-communicable diseases	8	3	10	3.20***
**Disabilities**	**109**	**117**	**107**	**0.91**
1. Hearing	36	40	34	0.85
2. Locomotion	33	35	32	0.91
3. Visual	39	39	39	1.00
4. Speech	2	2	1	0.61
**Accidents/injuries/poisoning**	**7**	**5**	**7**	1.56*
**Other diagnosed diseases**	**50**	**48**	**50**	**1.03**
**Other non-diagnosed diseases**	**16**	**17**	**16**	**0.90**
**Any Ailment^#^**	**328**	**299**	**337**	1.13***

Notes: ^#^Individual ailments will not add up to total because of reporting of multiple ailments.

1 includes Hepatitis/jaundice, amoebiosis, sexually transmitted diseases, malaria, eruptive, mumps, Diphtheria, filariasis/elephantiasis and others.

2 includes Neurological disorders, psychiatric disorders.

3 includes Prostatic disorders, gynecological disorders, goiter, tetanus, diseases of mouth/teeth/gum, cancer and other tumors, anaemia.

a Reference period of last 15 days prior to the survey.

@Gender gap = female/male, chi2>|z|: ***p<0.001, **p<0.05, *p<0.10.

In communicable disease category, prevalence of diarrhoea (6) and gastritis/gastric or peptic ulcer (21) followed by other communicable diseases (9) were significantly greater among older widows compared with older widowers. In contrast, the prevalence of whooping cough (10), skin diseases (9), and diseases of kidney/urinary system (8) were greater among older widowers. Tuberculosis (7) was next widely prevalent disease among older widowers.

The patterns of non-communicable diseases showed that prevalence of disorder of joints and bones (95), hypertension (63) and mellitus diabetes (30) diseases were significantly greater among older widows compared with older widowers followed by heart diseases (18), mental disorders (17) and eye diseases (15). This was contrasted by greater prevalence of cataract (54), bronchial asthma (43) and respiratory ailments (18) among older widowers. Not surprisingly, incidences of accidents/injuries/poisonings were more common among older widows higher by 56% (p<0.001) compared to older widowers. Furthermore, disability prevalence was comparatively greater, though statistically not significant among older widowers (117) than older widows (107).It is evident that the prevalence of non-communicable diseases is swiftly mounting in India coupled with the persistent communicable diseases. Consequently, older adults are at greater risk of reporting greater prevalence of acute chronic diseases, which are generally degenerative and human-made in nature. Results presented in this section depict significant gender differences in morbidity prevalence among older widowed adults. The disease burden is significantly greater among older widows compared to older widower. Patterns in morbidity prevalence indicate that older widows were reporting significantly greater prevalence of major chronic diseases such as diabetes, heart diseases, diarrhoea, mental illnesses, hypertension and other non-communicable diseases. In recent decades, a growing volume of literature has documented that India is facing a rapid pace of health-epidemiological transition with a swift increase in prevalence of chronic illness.

### Determinants of Morbidity Prevalence Rates

Table 2 presents effects of socio-economic and demographic factors on the likelihood of reporting various diseases in terms of communicable, non-communicable and other diseases estimated by fitting multinomial regression models. Adjusted percentages of older widows (60+) reporting specific type of ailments by different socio-economic and demographic background characteristics are arranged. Significant rural-urban differences were observed in morbidity prevalence among older widows. Older widows living in rural areas reported greater prevalence of communicable diseases and disabilities (16.8%) compared to those in urban areas (12.5%) and vice-versa in case of non-communicable diseases.

**Table 2:** Multinomial Logistic Regression Analysis: Adjusted Morbidity Prevalence (%) Among Older Widows (age 60+) by Socio-Demographic Background Characteristics in India, 2004.

Background variables	Communicable diseases	Non-communicable diseases	Other disease	Non-reporting
**Age**				
60–65 (rc)	5.10	17.88	8.11	68.91
65–70	4.29	24.17***	10.31***	61.24
70+	4.45	29.84***	14.65***	51.06
**Place of residence**				
Rural (rc)	5.58	21.48	11.12	61.82
Urban	3.28***	26.66***	9.29*	60.76
**Social group**				
Others (rc)	6.37	24.34	9.77	59.52
STs & SC	4.46***	21.45**	11.54	62.54
OBCs	4.14***	22.10**	10.90	62.87
**Religion**				
Hindu (rc)	4.81	22.31	10.69	62.20
Muslim	7.83***	29.32***	11.73**	51.13
Others	6.77***	30.73***	10.03	52.47
**Educational level**				
Illiterate (rc)	5.18	21.37	10.65	62.80
<Middle school complete	3.71	29.77***	10.74	55.78
Middle school complete	2.39	37.15***	8.92	51.55
High school complete & above	3.68	35.73***	10.82	49.77
**Living arrangement**				
Living alone (rc)	4.78	20.52	10.71	63.98
Living with children and other relatives	5.12	23.24**	10.39	61.26
Living with other non-relatives	3.46	21.26	13.21*	62.07
**Economic independence**				
Not dependent (rc)	3.89	20.29	9.65	66.17
Partially dependent	4.49	25.99***	8.05	61.47
Fully dependent	5.19**	22.74**	11.30**	60.77
MPCE@ **percentile class**				
Class1 (rc)	4.49	20.30	12.19	63.02
Class2	3.98	22.02	9.21***	64.79
Class3	5.09	21.72	10.61	62.59
Class4	5.56	22.35	10.74	61.35
Class5	5.22	26.40***	10.70	57.68
**Log likelihood**	**−6323.57**			
**LR χ2**	**653.03**			
**Prob.> χ2**	**0.001**			
**All**	**7.41**	**19.19**	**13.86**	**59.54**

Note: ***p<0.001, **p<0.05, *p<0.10, MPCE@- monthly per capita expenditure.

Reference category – rc.

The predictors age, economic independence, monthly per capita expenditure quintiles and education, showed positive direction of impact on the prevalence of ailments among older widows. The prevalence of non-communicable diseases increased significantly with age and the same pattern was observed for other types of diseases. Morbidity prevalence was greater among older widows in age 70+ (49%) compared with older widows in age (31%). The prevalence of morbidity increased with per capita expenditure percentile classes. Overall, 43% older widows of expenditure class5 reported ailments compared with 37% in expenditure class1. Non-communicable diseases were highly prevalent among older widows of monthly per capita expenditure percentile class5 (26.4%). However, no clear pattern was seen between expenditure classes and prevalence of communicable diseases and other diseases. Economically dependent older widows reported greater prevalence of morbidities (40%) compared with economically independent older widows (33%). Substantial education differentials were seen in the pattern of morbidity prevalence among older widows. The prevalence of communicable diseases was greater among illiterate older widows (5.2%) compared with high school & above pass older widows (3.7%). Contrary to this, older widows with high school & above education reported significantly greater prevalence of non-communicable diseases (35.7%) compared with illiterate older widows (21.4%).

By religion, older widows of Hindu religion reported lower prevalence of both communicable and non-communicable morbidities compared with Muslims and others. A lower prevalence of non-communicable diseases was reported among older widows belonging to Hindu religion (22.3%) compared with Muslims (29.3%) and others (30.7%). The prevalence of communicable diseases was also lower among older widows of Hindu religion (5%) compared to Muslims (7.8%) and Others (6.8%). Non-communicable diseases were highly prevalent among older widows of general caste (24.3%) compared to scheduled caste/scheduled tribes (21.4%) and other backward classes older widows (22.1%). At the same time, prevalence of communicable diseases (6.4%) was also significantly greater among older widows of general caste groups. Results by living arrangement showed that older widows living with their relatives/non-relatives reported greater prevalence of diseases compared with those living alone. However, disease pattern showed that the prevalence of communicable diseases was greater among older widows living alone (4.8%) compared those living with non-relatives (3.5%).

### Health Care Seeking Behavior

The adjusted odd ratios from logistic regression analysis on the likelihood of utilizing health care services among those older widows who reported morbidities by socio-economic and demographic determinants are presented in Table 3. Results showed that older widows living in urban areas had 14% greater likelihood of accessing health care services compared to older widows living in rural areas at 10% level of significance. Age was negatively associated with utilization of health care services, particularly among oldest-old widows. Oldest-old widows in age 70+ were 15% (p<0.10) less likely to seek health care services compared with older widows in age 60–65.

**Table 3:** Logistic Regression Analysis: Modelling of Socio-economic and Demographic Determinants of Health Care Seeking Behavior Among Older Widows (60+) With Morbidities in India, 2004.

Background Variables	Exp(β)	(95% CI)
**Place of residence (ref. = rural)**		
Urban	1.14*	(0.97–1.35)
**Age(ref. = 60–65)**		
65–70	1.04	(0.85–1.28)
70+	0.85*	(0.71–1.02)
**Religion(ref. = Hindu)**		
Muslim	1.08	(0.87–1.34)
Others	1.02	(0.77–1.35)
**Social Group(ref. = others)**		
STs & SC	0.58***	(0.48–0.71)
OBCs	0.74***	(0.63–0.88)
**Educational Level(ref. = illiterate)**		
<Middle school complete	1.62***	(1.28–2.06)
Middle school complete	3.55***	(1.89–6.68)
High school complete and above	3.84***	(1.83–8.04)
**Living Arrangement(ref. = living alone)**		
Living with children and other relatives	1.49***	(1.17–1.89)
Living with other non-relatives	1.14	(0.83–1.57)
**Economic Independence(ref. = not dependent)**		
Partially Dependent	1.10	(0.82–1.49)
Fully Dependent	1.12	(0.88–1.42)
MPCE@ **percentile class (ref. = Class1)**		
Class2	1.28***	(1.00–1.63)
Class3	1.65***	(1.30–2.09)
Class4	2.09***	(1.65–2.65)
Class5	2.87***	(2.22–3.71)
**Log likelihood**	**−2396.97**	
**LR χ2**	**362.7**	
**Prob.> χ2**	**0.001**	

Note: The sample for analysis of utilization of health care services is those older widows who reported morbidities; MPCE = monthly per capita expenditure.

***p<0.001, **p<0.05, *p<0.10.

No significant association was observed between religion and health care use among older widows. Older widows of scheduled caste/tribes and other backward classes were significantly less likely to seek treatment compared with older widows of other/general caste groups respectively by 42% (p<0.001) and 26% (p<0.001). The likelihood of seeking health care services increased significantly with the level of education. Older widows literate up to middle school were 1.6 (p<0.001) times more likely to seek treatment compared with illiterates. Similarly compared to illiterates, older widows with middle pass and high school & above education were more likely to seek treatment respectively by 3.6 (p<0.001) and 3.8 times (p<0.001).

It is indeed that better economic conditions positively influence the likelihood of utilizing health care services. A strong positive relation was observed between monthly per capita expenditure quintiles and health care utilization among older widows. Older widows of MPCE class5 were 2.9 times (p<0.001) more likely to seek treatment for reported morbidities compared with older widows of MPCE class1. On the other hand, economically dependent older widows had greater likelihood of seeking treatment for the reported ailments. This could be possible due to the fact that majority of economically independent older widows were living alone and at the same time, there was no source of income for them.

Living arrangement is most plausible factor for the treatment seeking behavior among older widows. Older widows living with children and other relatives had 49% (p<0.001) greater likelihood of seeking health treatment for reported diseases compared with those living alone. Similarly, older widows living with non-relatives were having 14%, though statistically not significant, higher chances of seeking health care services compared with older widows living alone.

## Discussion and Conclusion

So far, no attempts have been made to study the disease patterns among older widows and their treatment seeking behavior in India. At the same time, very limited information on the health conditions of older widows and their treatment seeking behavior is available. In a very first effort, this paper congregated critical evidences that older widows suffered with greater rates of self-reported morbidities and a very lower proportion of older widows were able to access health care services. Disease patterns showed that non-communicable disease were more widely prevalent among older widows. On the other hand, contribution of communicable diseases to disease burden was comparatively lower. These patterns in disease prevalence were in expected direction as India is swiftly entering in the advanced phases of health transition and demographic ageing [10]. Here, the most striking concern was emerged that a greater proportion of older widows reported life-style, behavioural and environmental related morbidities compared to their counterparts, older widowers.

Substantial disparities in disease prevalence patterns and treatment seeking behaviour were noticed by age, residence, education and other socio-economic conditions. Oldest-old widows reported greater prevalence of morbidities due to weakening resistance power in old ages [39]–[43]. Better socio-economic status is strongly associated with better self-reporting of health status and greater utilization of health care services [9], [18], [44]–[49]. However, in the process of health-epidemiological transition, higher socio-economic status is also associated with the greater reporting of sedentary life style related morbidities. Likewise, older widows with low socio-economic status reported greater prevalence of communicable diseases and vice-versa for non-communicable diseases.

There were strong reasons to assess rural-urban differences in health and well-being conditions of older widows particularly in Indian traditional societies. First, major chunk of older widow’s population lives in rural India (75%). Second, widows from rural areas tend to be highly marginalised person in terms of socio-economic conditions due to various patriarchal norms such as patriarchal inheritance and division of labour by gender coupled with the lack of social reforms for older widows in rural India [28], [30]. Third, better quality health care services are more concentrated in urban areas and, still to achieve in rural areas even for general population. Fourth, due to long period social negligence, older widows are most likely to perceive their ill-health condition as god-gifted.

The consequences of this social and economic marginalisation were manifest in poor health conditions and low levels of health care services utilization. Older widows in rural areas reported greater prevalence of communicable diseases and lesser utilization of health care services. This is plausible as older widows living in rural areas are more prone to poor household environmental conditions such as use of solid fuel for cooking, poor access to water and sanitation facilities. The government did not incline to give adequate priority to the social protection of widows in rural India in the absence of reliable and adequate information on health conditions of older widows. An effective implementation of social security measures may require a great deal of activism on the part of non-government institutions, including the women’s movement, particularly in rural areas [28].

This study has important policy implications too. Overall, marital status coupled with age plays a significant role in determination of health and the relationship we investigated is sensitive for gender too and therefore, the health policy should take care of vulnerable groups in a particular stage of life [36]. Given the evolving scenario of ageing, particularly its female dimension, questions of support and care to the female aged especially when they are widows need to be addressed first [2]. At present, India is having a national policy for older persons [50]. However, how effectively it is implemented in the last decade, is a big question.

The present policy need to be reformulated to come out with a comprehensive policy for older persons. The policy should address the socio-economic aspects of older persons and proper attention should be given to most vulnerable groups of older persons such as older widows living in rural areas and those are socially and economically backward. Community level interventions are urgently required to spread awareness and knowledge among older persons particularly those with low socio-economic conditions [10], [22], [47].

Last but not the least, there is need for a prevention strategy that may include lifestyle changes during middle age in order to curtail the incidence or at least severity of life-style related morbidities that are reported by older widows with better socio-economic status [10], [23], [51].

## Supporting Information

Appendix Table S1 **Percentage distribution of Widowed Older Persons (age 60 and above) by Various Socio-demographic Categories in India, 2004.**
(DOCX)
