# Supplementary material for: Morbidity Patterns and Health Care Seeking Behavior among Older Widows in India
Source: PLoS One. 2014 Apr 9;9(4):e94295. doi: 10.1371/journal.pone.0094295 (PMC3981780; doi:10.1371/journal.pone.0094295)
Supplement: Appendix Table S1 — Percentage distribution of Widowed Older Persons (age 60 and above) by Various Socio-demographic Categories in India, 2004. (DOCX) [file pone.0094295.s001.docx]

**Table S1 Percentage distribution of Widowed Older Persons (age 60 and above) by Various Socio-demographic Categories in India, 2004**

| **Background Characteristics** | **Total** | **Male** | **Female** |
| --- | --- | --- | --- |
| **Place of residence** | |  |  |
| Rural | 76.3 | 79.8 | 75.2 |
| Urban | 23.7 | 20.2 | 24.8 |
| **Social group** | |  |  |
| Others | 33.9 | 31.9 | 34.6 |
| SC/ST | 25.0 | 25.9 | 24.7 |
| OBC | 41.1 | 42.2 | 40.8 |
| **Educational level** | |  |  |
| Illiterate | 79.8 | 62.3 | 85.2 |
| <Middle class | 13.8 | 23.4 | 10.8 |
| Middle class | 3.5 | 7.5 | 2.2 |
| High school & above | 3.0 | 6.9 | 1.8 |
| **Religion** |  |  |  |
| Hindu | 85.0 | 86.5 | 84.5 |
| Muslim | 9.7 | 8.4 | 10.2 |
| Others | 5.3 | 5.2 | 5.3 |
| **Living arrangement** | |  |  |
| Living alone | 11.9 | 10.1 | 12.4 |
| Living with children & other members | 79.3 | 81.7 | 78.6 |
| Living with others | 8.8 | 8.2 | 9.0 |
| **Economic independence** | | |  |
| Not dependent | 19.6 | 35.3 | 14.7 |
| Partially dependent | 11.4 | 13.2 | 10.8 |
| Fully dependent | 69.0 | 51.5 | 74.5 |
| **MPCE^@^ percentile class** |  |  |  |
| Class1 | 16.7 | 16.2 | 16.9 |
| Class2 | 18.6 | 21.2 | 17.8 |
| Class3 | 19.8 | 19.6 | 19.8 |
| Class4 | 22.4 | 23.5 | 22.1 |
| Class5 | 22.5 | 19.6 | 23.3 |
| **Age** |  |  |  |
| 60-65 | 26.7 | 24.3 | 27.5 |
| 65-69 | 28.3 | 24.4 | 29.5 |
| 70+ | 45.0 | 51.3 | 43.1 |
| **Total** | **100.0** | **23.7** | **76.3** |
| **N** | **13278** | **3267** | **10111** |
